# Progress Toward Global Eradication of Dracunculiasis, January 2019–June 2020

**DOI:** 10.15585/mmwr.mm6943a2

**Published:** 2020-10-30

**Authors:** Donald R. Hopkins, Adam J. Weiss, Sharon L. Roy, Sarah Yerian, Sarah G.H. Sapp

**Affiliations:** ^1^The Carter Center, Atlanta, Georgia; ^2^Division of Parasitic Diseases and Malaria, Center for Global Health, World Health Organization Collaborating Center for Dracunculiasis Eradication, CDC.

Dracunculiasis (Guinea worm disease) is caused by the parasite *Dracunculus medinensis* and is acquired by drinking water containing copepods (water fleas) infected with *D. medinensis* larvae. The worm typically emerges through the skin on a lower limb approximately 1 year after infection, resulting in pain and disability (*1*). There is no vaccine or medicine to treat the disease; eradication efforts rely on case containment* to prevent water contamination. Other interventions to prevent infection include health education, water filtration, chemical treatment of unsafe water with temephos (an organophosphate larvicide to kill copepods), and provision of safe drinking water (*1*,*2*). The worldwide eradication campaign began in 1980 at CDC *(**1**)*. In 1986, with an estimated 3.5 million cases^†^ occurring each year in 20 African and Asian countries^§^ (*3*), the World Health Assembly (WHA) called for dracunculiasis elimination (*4*). The global Guinea Worm Eradication Program (GWEP), led by the Carter Center and supported by the World Health Organization (WHO), United Nations Children’s Fund, CDC, and other partners, began assisting ministries of health in countries with dracunculiasis. This report, based on updated health ministry data (*4*), describes progress made during January 2019–June 2020 and updates previous reports (*2*,*4*,*5*). With only 54 human cases reported in 2019, 19 human cases reported during January 2019–June 2020, and only six countries currently affected by dracunculiasis (Angola, Chad, Ethiopia, Mali, South Sudan, and importations into Cameroon), the achievement of eradication is within reach, but it is challenged by civil unrest, insecurity, and lingering epidemiologic and zoologic concerns, including 2,000 reported animal cases in 2019 and 1,063 animal cases in 2020, mostly in dogs. All national GWEPs remain fully operational, with precautions taken to ensure safety of program staff members and community members in response to the coronavirus disease 2019 (COVID-19) pandemic.

In March 2019, the Carter Center hosted the annual GWEP Managers Meeting in Atlanta, Georgia, WHO’s International Commission for the Certification of Dracunculiasis Eradication (ICCDE) met in Addis Ababa, Ethiopia, in April 2019, and WHO convened the annual Informal Meeting of Ministers of Health of endemic and formerly endemic dracunculiasis-affected countries during the WHA in Geneva, Switzerland, in May 2019. Because of the COVID-19 pandemic, The Carter Center hosted the annual GWEP Managers’ Meeting and a meeting of Guinea worm researchers virtually in March 2020; WHO’s ICCDE did not meet, and the Informal Meeting during the WHA did not occur during the first half of 2020. WHO has certified 199 countries, areas, and territories as free from human dracunculiasis, with only seven countries still lacking certification (*4*).

In 2019, Angola, Cameroon, Chad, and South Sudan reported 54 human cases; Angola, Chad, Ethiopia, and Mali reported 2,000 infected animals (mostly dogs), compared with 28 human cases and 1,102 animal infections reported in 2018 (Table 1). During January–June 2020, human cases were reported in Chad (nine), Ethiopia (seven), Mali (one), Cameroon (one), and Angola (one), with 1,063 infected animals reported, compared with 31 human cases and 1,365 infected animals reported during January–June 2019. During January–June 2020, CDC received 44 specimens from humans for morphologic or molecular identification, including 21 (41%) that were laboratory-confirmed as *D. medinensis*,^¶^ compared with 127 specimens received and 67 (53%) confirmed during all of 2019 (Table 2). During the first 6 months of 2020, CDC received 19 specimens from animals, five (26%) of which were confirmed *D. medinensis*, compared with 59 received and 33 (56%) confirmed during all of 2019. Two specimens from unknown host sources during 2019 were not *D. medinensis*. *D. medinensis* worms removed from animals are genetically and morphologically indistinguishable from those removed from humans (*6*).

**TABLE 1:** Number of reported indigenous dracunculiasis cases, by country –– worldwide, January 2018–June 2020

Country	No (% contained)		% Change Jan–Dec 2018 to Jan–Dec 2019	No. (% contained)		% Change Jan–Jun 2019 to Jan–Jun 2020
	Jan–Dec 2018	Jan–Dec 2019		Jan–Jun 2019	Jan–Jun 2020	
	No. (% contained)	No. (% contained)		No. (% contained)	No. (% contained)	
**Human cases**						
Chad	17 (41)	48 (54)	188	29 (59)	9 (44)	−69
Ethiopia	0	0	0	0	7 (100)	NA
Mali*	0	0	0	0	1 (0)	NA
South Sudan	10 (30)	4 (50)	−60	0 (0)	0	NA
Angola	1 (0)	1 (0)	0	1 (0)	1 (0)	0
Cameroon	0	1 (0)**^†^**	0	1 (0)**^†^**	1 (0)^§^	0
**Total**	**28 (36)**	**54 (52)**	**93**	**31 (55)**	**19 (58)**	−**39**
**Animal infections** ^¶^						
Chad	1,065 (75)	1,982 (76)	82	1,356 (78)	1,057 (87)	−22
Ethiopia	17 (41)	8 (25)	−53	6 (0)	3 (33)	−50
Mali	20 (80)	9 (67)	−55	2 (100)	0	−100
Angola	0	1 (0)	NA	1 (0)	0	−100
Cameroon	0	0	0	0	3 (0)	NA
**Total**	**1,102 (75)**	**2,000 (76)**	**77**	**1,365 (78)**	**1,063 (87)**	−**22**

**Abbreviation:** NA = not applicable.

* Civil unrest and insecurity since a coup d'état in April 2012 continued to constrain program operations in regions with endemic dracunculiasis (Gao, Kidal, Mopti, and Timbuktu) during 2019–June 2020.

**^†^** One case was reported from Cameroon in 2019 in a village approximately 1 mile (1.5 km) from the Chad-Cameroon border. This is believed to have been acquired in Chad.

^§^ One human case and three infected dogs detected in an area of Cameroon near the border with Chad in February–March 2020 might have also been infected in Chad.

^¶^ In Chad, primarily dogs, some cats; in Ethiopia, dogs, cats, and baboons; in Mali, dogs and cats; in Angola, one dog.

**TABLE 2:** Characteristics of specimens from humans and animals received at CDC for laboratory diagnosis of *Dracunculus medinensis* — January 2019–June 2020

Specimens received at CDC	Jan–Dec 2019	Jan–Jun 2020
**Specimens from humans**		
No. received	127	44
**No. (%) laboratory confirmed as *D. medinensis***	67 (53)	21 (41)
**Country of origin, no. of specimens (no. of patients)**		
Angola	1 (1)	1 (1)
Cameroon	1 (1)	1 (1)
Chad	50 (49)	9 (9)
Ethiopia	—	9 (7)
Mali	—	1 (1)
South Sudan	15 (4)	—
**No. (%) ruled out as *D. medinensis***	60 (47)	23 (59)
**No. (%) of other laboratory diagnoses**		
Free-living nematode*	7 (12)	2 (9)
*Onchocerca*	3 (5)	1 (4)
Other parasitic nematode*	2 (3)^†^	3 (13)^†^
Sparganum	20 (33)	8 (35)
Tissue	8 (13)	2 (9)
Plant material	4 (7)	—
Other worms	1 (2)^§^	4 (17)^§^
Other	—	1 (4) ^⁋^
Unknown origin	15 (25)	2 (9)
**Specimens from animals**		
No. received	59	19
**No. (%) laboratory confirmed as *D. medinensis***	33 (56)	5 (26)
**Country/Species of origin, no. of specimens (no. of animals)**		
Angola	3	—
Dog	3 (1)	—
Cameroon	—	3
Dog	—	3 (100)
Chad	16	—
Cat	2 (2)	—
Dog	14 (14)	—
Ethiopia	5	2
Baboon	1 (1)	2 (100)
Leopard	1 (1)	—
Dog	3 (3)	—
Mali	9	—
Cat	1 (1)	—
Dog	8 (8)	—
**No. (%) ruled out as *D. medinensis***	26 (44)	14 (74)
**No. (%) of other laboratory diagnoses**		
Free-living nematode*	11 (42)	—
Other parasitic nematode*	12 (46)**	11 (78)**
Tissue	—	1 (7)
Other worms	1 (4)^††^	1 (7)^††^
Other	—	1 (7)^§§^
Unknown origin	2 (8)	0
**Specimens from unknown sources**		
No. received	2	—
**No. (%) laboratory confirmed as *D. medinensis***	0	—
**No. (%) ruled out as *D. medinensis***	2 (100)	—
**No. (%) of other laboratory diagnoses**		
Free-living nematode	2 (100)	—

* Free-living nematodes primarily included adult Mermithidae and other nematodes identified as belonging to nonparasitic taxa. Other parasitic nematodes included non-*Onchocerca* nematodes identified as belonging to parasitic taxa.

^†^ Other parasitic nematodes submitted in association with human cases in 2019 included *Elaeophora* sp. (one) and a filarial nematode not identified to genus (one); during January–June 2020 submissions included *Dirofilaria* sp. (one), *Eustrongylides* sp. (one), and nematodes not identified further (two).

^§^ Other worms submitted in association with a human case in 2019 included a single tapeworm not identified further. Submissions in this category from human cases during January–June 2020 included an annelid (one); a horsehair (Gordian) worm (one); a specimen vial that contained two Acanthocephala not identified further and one *Toxocara*; and one nematode not able to be identified further (one).

^⁋^ The other specimen submitted in association with a human case during January–June 2020 was a small (approximately 15 cm) blind snake (infraorder Scolecophidia).

** Other parasitic nematodes submitted in association with animal cases in 2019 included *Dirofilaria* sp. (one), *Eustrongylides* sp. (three), *Filaria* sp. (one), *Physaloptera* sp. (one), *Spirura* sp. (one), *Setaria* sp. (one), and filarial nematodes not identified to genus (three); during January–June 2020, submissions included *Protospirura* sp. (one), *Setaria* sp. (one), *Skrjabinodera* sp. (two), filarial nematodes not identified to genus (six), and a spirurid nematode not identified to genus (one).

^††^ Other worms submitted in association with an animal case included a *Taenia* sp. in 2019, and an Acanthocephala not identified further during January–June 2020.

^§§^ The other specimen submitted in association with an animal case during January–June 2020 was a small blind snake (infraorder Scolecophidia).

In affected countries, the national GWEP receives monthly case reports from supervised volunteers in each village under active surveillance** (Table 3). Villages where endemic transmission has ended (i.e., zero human cases or animal infections reported for ≥12 consecutive months) are kept under active surveillance for 2 additional years. WHO certifies a country as dracunculiasis-free after adequate nationwide surveillance for ≥3 consecutive years with no indigenous human cases or animal infections.^††^

**TABLE 3:** Reported human and animal dracunculiasis cases, surveillance, and status of local interventions in villages with endemic disease, by country—worldwide, 2019

Human cases/Surveillance/Intervention status	Country					
	Chad*	Ethiopia	Mali^†^	South Sudan	Angola	Total
**Reported human cases**						
No. indigenous, 2019	49^§^	0	0	4	1	**54**
No. imported,^¶^ 2019	0	0	0	0	0	**0**
% Contained** in 2019	53	0	0	50	0	**52**
% Change in indigenous human cases in villages/localities under surveillance, same period 2018 and 2019	188	0	0	−60	0	**93**
**Reported animal cases**						
No. indigenous, 2019	1,935	8	6	0	1	**1,950**
No. imported,^††^ 2019	0	0	3	0	0	**3**
% Contained** in 2019	77	25	67	0	0	**77**
% Change in indigenous animal cases in villages/localities under surveillance, same period 2018 and 2019	82	−53	−55	0	NA	**77**
**Villages under active surveillance, 2019**						
No. of villages	2,211	189	2,802	2,675	0	**7,877**
% Reporting monthly	97	100	100	88	0	**96**
No. reporting ≥1 human case	25	0	0	10	1	**38**
No. reporting only imported^††^ human cases	0	0	0	0	0	**0**
No. reporting indigenous human cases	25	0	0	10	1	**38**
No. reporting ≥1 animal case	422	4	8	2	0	**436**
No. reporting only imported^††^ animal cases	0	0	2	0	0	**2**
No. reporting indigenous animal cases	422	4	6	2	0	**434**
**Status of interventions in villages with endemic human dracunculiasis, 2019**						
No. of villages with endemic human dracunculiasis, 2018–2019	34	0	0	12	2	**48**
% Reporting monthly^§§^	100	NA	NA	87	—	**92**
% Filters in all households^§§^	20	NA	NA	58	—	**29**
% Using temephos^§§^	61	NA	NA	75	—	**63**
% ≥1 source of safe water^§§^	50	NA	NA	67	100	**52**
% Provided health education^§§^	100	NA	NA	92	100	**94**
**Status of interventions in villages with endemic animal dracunculiasis, 2019**						
No. of villages with endemic animal dracunculiasis, 2018–2019	526	11	22	0	1	560
% Reporting monthly^§§^	100	100	100	NA	—	100
% Using temephos^§§^	69	100	100	NA	—	70
% Provided health education^§§^	100	100	100	NA	100	100

**Abbreviation:** NA = not applicable.

* Participants at the annual Chad Guinea Worm Eradication Program review meeting in November 2014 adopted “1+ case village” as a new description for villages in Chad affected by human cases of Guinea worm disease and/or dogs infected with Guinea worms and defined it as “a village with one or more indigenous and/or imported cases of Guinea worm infections in humans, dogs, and/or cats in the current calendar year and/or previous year.”

^†^ Civil unrest and insecurity since a coup in 2012 continued to constrain Guinea Worm Eradication Program operations (supervision, surveillance, and interventions) in Gao, Kidal, Mopti, Segou, and Timbuktu Regions.

^§^ Forty-eight cases were reported from Chad in 2019. One case was reported from Cameroon in 2019 in a village approximately 1 mile (1.5 km) from the Chad-Cameroon border. This is believed to have been acquired in Chad.

^¶^ Imported from another country.

** Transmission from a patient with dracunculiasis is contained only if all of the following conditions are met for each emerged worm: 1) the infected patient is identified ≤24 hours after worm emergence; 2) the patient has not entered any water source since the worm emerged; 3) a village volunteer or other healthcare provider has managed the patient properly, by cleaning and bandaging the lesion until the worm has been fully removed manually and by providing health education to discourage the patient from contaminating any water source (if two or more emerging worms are present, transmission is not contained until the last worm is removed); 4) the containment process, including verification of dracunculiasis, is validated by a Guinea Worm Eradication Program supervisor within 7 days of emergence of the worm; and 5) temephos is used to treat potentially contaminated surface water if any uncertainty about contamination of these sources of drinking water exists, or if a such a source of drinking water is known to have been contaminated.

^††^ Imported from another in-country disease-endemic village.

^§§^ The denominator is the number of endemic villages/localities where the program applied interventions during 2018–2019.

## Country Reports

**Angola.** Before 2018 no case of dracunculiasis was ever reported from Angola. After the discovery of a case in a human with no history of foreign travel in Cunene Province in April 2018, Angolan health authorities and WHO investigated nearby communities and began training local health professionals and community health workers about the disease (*4*) but found no other active cases. Another case was detected in January 2019 and a third in March 2020, both in Cunene Province in persons with no foreign travel. In April 2019, a dog with an emerging Guinea worm was found in the same district as were the first and third human cases. Angola offers a US$450 equivalent cash reward for reporting an infected human or animal. Provisional DNA analysis of Angola’s Guinea worm specimens yielded no clear link to another *D. medinensis* population.

**Chad**. Chad reported 48 cases in 25 villages in 2019. During the first half of 2020, Chad reported nine human cases in seven villages (including four new villages), compared with 29 cases during January–June 2019 (Table 1). Twenty-two of the cases reported in 2019 were associated with one village in Salamat Region, representing Chad’s first documented waterborne outbreak of dracunculiasis in humans since 2010. A Cameroonian woman whose village is approximately 1 mile (1.5 km) from the Chad-Cameroon border had a Guinea worm emerge in 2019; she was likely infected in Chad, as were one human and three dogs in the same area of Cameroon through June 2020.

During 2019, 1,935 domestic dogs and 47 domestic cat infections were reported, nearly twice the 1,040 dog and 25 cat infections reported in 2018. During January–June 2020, 24% fewer infected dogs and 56% more infected cats were reported than were during January–June 2019. The Carter Center is helping the Chad Ministry of Health implement active village-based surveillance in 2,219 at-risk villages (as of June 2020), compared with 2,211 villages in December 2019. The working hypothesis is that humans, dogs, and cats might become infected by eating inadequately cooked fish or other aquatic transport or paratenic hosts (hosts in which the larval parasite does not develop) (*7*). Since June 2017, approximately 81% of households sampled monthly in at-risk communities were burying fish entrails as recommended; 77% and 87% of infected dogs were tethered (contained) in 2019 and during January–June 2020, respectively. Temephos application reached 68% of 422 villages with dog or human infections by December 2019 and 73% by April 2020. In December 2019, 65% of villages reporting infected dogs or humans had at least one source of copepod-free drinking water.

In areas under surveillance in Chad, 59% of residents surveyed in 2019 knew of the cash rewards for reporting a human (US$100 equivalent) or animal (US$20 equivalent) infection, and during January–June 2020, 86% knew of the rewards. Intensified surveillance generated 50,893 rumors (reports about a possible Guinea worm infection) regarding human or dog infections during January–June 2020 compared with 41,501 rumors during the same period in 2019; a person or dog with compatible signs or symptoms is suspected of having dracunculiasis, pending confirmation.

**Ethiopia**. Ethiopia reported no human case during 2018–2019 but reported seven during January–June 2020. The 2020 cases were in villagers exposed to a shared source of contaminated drinking water near Duli village in Gambella Region. During 2019, Ethiopia reported two infected dogs and six infected baboons, all in Gog district of Gambella Region, compared with 17 infected animals (11 dogs, five cats, and one baboon) in 2018. During January–June 2020, Ethiopia reported one infected dog and two infected baboons, all in Gog district, compared with six infected baboons in January–June 2019. Since 2017, The Carter Center has supported Ethiopian public health and wildlife authorities in a baboon and dog epidemiology project (*2*).

The Ethiopia Dracunculiasis Eradication Program has 189 villages under active surveillance. It applies temephos monthly to almost all water sources known to have been used by humans in the at-risk area of Gog district, and since 2018 it has supported villager-initiated, constant tethering of approximately 1,100 dogs and cats in villages where most infected animals were detected in recent years to prevent their exposure to water sources in adjacent forests where transmission is believed to occur (*2*). In 2018, Ethiopia increased its rewards for reporting a human dracunculiasis case to US$360 equivalent and for reporting and tethering an infected animal to US$40. In 2019, 74% and 96% of persons surveyed in active surveillance areas were aware of the rewards for reporting infected humans and animals, respectively.

**Mali.** In 2019, Mali reported no human dracunculiasis case for the fourth consecutive year; one case was reported during January–June 2020. During 2019, eight infected dogs and one infected cat were reported, compared with 18 dogs and two cats in 2018. During the first half of 2020, Mali reported no infected dog or cat, compared with two infected dogs during the first half of 2019. Six of the nine infected animals identified in 2019 were detected in Segou Region; three dogs were detected in adjacent Djenne district of Mopti Region. Segou Region is accessible to the program, but the dogs were bred and apparently became infected in areas of Mopti Region that have not been accessible to the program since 2012 because of insecurity; some dogs were later sold in Segou. The infected human in 2020 was detected in Segou Region. In 2019, Mali increased the number of villages under active surveillance to 2,802 from 903 at the end of 2018 and increased the cash reward to US$340 equivalent for reporting a case in a human; the reward remains at US$20 equivalent for reporting and tethering an infected animal. In areas under active surveillance, 77% of persons queried in 2019 and 86% in January–June 2020 were aware of the cash rewards for reporting an infected person or animal.

**South Sudan.** South Sudan reported four human cases in 2019, compared with 10 in 2018 and no human cases in January–June 2020 or in January–June 2019. Only one infected animal was reported in 2015, a dog in the same household as an infected person. Extreme population mobility of cattle herders and others is a special challenge in addition to sporadic insecurity. By December 2019, South Sudan’s Guinea Worm Eradication Program had 2,675 villages under active surveillance. The cash reward for reporting a case of dracunculiasis is about US$400 equivalent. A 2019 survey of residents in villages not under active surveillance found that 73% of the respondents knew of the reward for reporting an infected person.

## Discussion

Chad reported 3,096 (99%) of the world’s 3,136 *D. medinensis* infections reported during January 2019–June 2020, 95% of which were in dogs. After a decade with no reported cases, Chad reported 10 indigenous human cases in 2010. Guinea worm infections in domestic dogs were reported for the first time in 2012, mostly from communities along the Chari River (*7*). Stopping transmission among dogs in Chad is now the biggest challenge faced by the eradication program. It is being addressed through innovative interventions and research supported by The Carter Center, WHO, and CDC and involves multiple research institutions with the purpose of better understanding the unusual epidemiology of dracunculiasis in the remaining countries with endemic transmission and assessing antihelminthic treatment of dogs (*8*). For example, collaboration with researchers from the University of Georgia (Athens, Georgia) has shown that fish can serve as transport hosts for *Dracunculus* spp. in the laboratory and that *D. medinensis* can use frogs as paratenic hosts; *Dracunculus* larvae have been recovered from multiple wild frogs in Chad (*9*,*10*). Common source waterborne dracunculiasis outbreaks in Chad in 2019 and Ethiopia in 2020 highlight the need for safe drinking water wherever this disease occurs.

Chad’s ministry of health has offered a US$100 equivalent reward for reporting a confirmed human dracunculiasis case since 2010 and a reward of US$20 for reporting and tethering an infected dog since 2015. The rewards are given only after a case is confirmed; all reports must be corroborated by supervisors. In 2017, Chad launched a nationwide communication campaign to increase awareness about the rewards and how to prevent dracunculiasis in humans and dogs. Since 2013, Chad’s GWEP has urged villagers to cook their fish well, bury fish entrails, and prevent animals from eating them. In 2014, village volunteers began persuading villagers to tether infected dogs until the worms emerged to prevent contamination of water. In March 2020, the program launched a new strategy to tether dogs proactively during the 4 months of peak dracunculiasis incidence in the 118 villages that reported five or more dracunculiasis infections in 2019. The program began applying temephos to cordoned sections of the extremely large lagoons at entry points used by infected humans or dogs in 2014, and it began applying temephos monthly to small ponds in villages with the most infected dogs in 2017.

The pattern of transmission to many dogs and few humans in Chad remains peculiar to that country. If the hypothesis is correct that the parasite’s life cycle in Chad involves a transport or paratenic host (*10*), increased active surveillance, containment of infected dogs, application of temephos, and burial of fish entrails should reduce transmission. The dracunculiasis cases found in Cameroon in 2019 and 2020 highlight the risks for cases exported from Chad and the need for ongoing active surveillance in neighboring countries, especially Cameroon and the Central African Republic.

Finding three confirmed cases in humans and one infected dog in Angola during 2018–2020 suggests that the problem there is limited, but active surveillance throughout the at-risk areas is required to determine its full extent. If adequate security is maintained, South Sudan is poised to achieve zero-case status soon based on strong technical leadership, strong governmental political support, and no parallel animal infections.

In 2020, Mali reported its first human case in approximately 4 years, and Ethiopia reported its first human cases in approximately 2 years. Continued endemic transmission of Guinea worm infections among a few dogs and cats in Mali as well as baboons in Ethiopia appears to be geographically limited in each country. The ecologic study of baboons and proactive tethering of dogs in Gog district might help the program understand the dynamics of residual Guinea worm infections in Ethiopia. Insecurity decreased in some Guinea worm–affected areas of Mali in 2019 and 2020 but is still the main obstacle to stopping transmission in that country.

**Summary**

**What is already known about this topic?**

Human dracunculiasis (Guinea worm disease) cases have decreased from an estimated 3.5 million in 1986 to 54 in 2019. Guinea worm infection in dogs has complicated eradication efforts.

**What is added by this report?**

During January–June 2020, the number of human dracunculiasis cases reported decreased to 19 in four countries (Angola, Chad, Ethiopia, and Mali) with one case in Cameroon in a patient possibly infected in Chad; South Sudan reported no human cases. In addition, 1,063 infected animals were reported

**What are the implications for public health practice?**

Infected dogs, especially in Chad, and impeded access because of civil unrest and insecurity in Mali and South Sudan remain challenges to interrupting transmission.

## Footnotes

* Transmission from a patient with dracunculiasis is considered contained only if all of the following conditions are met for each emerging worm: 1) the infected patient is identified ≤24 hours after worm emergence; 2) the patient has not entered any water source since the worm emerged; 3) a village volunteer or other health care provider has managed the patient properly; 4) the containment process, including verification of dracunculiasis, is validated by a Guinea Worm Eradication Program supervisor within 7 days of emergence of the worm; and 5) the approved chemical temephos (Abate) is used to treat known or potentially contaminated surface water. Proper patient management includes cleaning and bandaging the lesion until the worm has been fully removed manually and by providing health education to discourage the patient from contaminating any water source. If two or more emerging worms are present, transmission is not contained until the last worm is removed. Similar criteria are in place for the containment of animal infections.

† A dracunculiasis case is defined as an infection occurring in a person exhibiting a skin lesion or lesions with emergence of one or more worms laboratory-confirmed at CDC as *D. medinensis*. Because *D. medinensis* has a 10- to 14-month incubation period, each infected person is counted as having a case only once during a calendar year.

§ Initially 20 countries, but the former country of Sudan officially separated into two countries (Sudan and South Sudan) on July 9, 2011.

¶ Specimens are laboratory-confirmed as *D. medinensis* at CDC by morphologic examination under a microscope or polymerase chain reaction assay. Additional information about laboratory identification of parasites is available at https://www.cdc.gov/dpdx/dxassistance.html.

** Villages under active surveillance are those that have endemic dracunculiasis or are at high risk for importation. Active surveillance involves daily searches of households by village volunteers (supported by their supervisors) for persons or animals with signs of dracunculiasis. An imported human case or animal infection is one resulting from ingestion of contaminated water in a place other than the community where the case or infection is detected and reported. Since 2012, no internationally imported cases or infections have been reported.

†† An indigenous dracunculiasis human case or animal infection is defined as an infection consisting of a skin lesion or lesions with emergence of one or more Guinea worms in a person or animal who had no history of travel outside their residential locality during the preceding year.
